# A Plea for Co-Operative Homes for Workers

**Published:** 1918-11-23

**Authors:** 


### THE HOUSING PROBLEM.

### A Plea for Co-operative Homes for Workers.

"One of the matters that will first have to be considered after the war is the question of providing suitable dwellings for our working men." Statements such as this appear almost daily in the press, and various estimates of what a proper house for a working-class family ought to be. A recent estimate was a parlour, a large kitchen living-room, a scullery, three rooms over the top of these, and certain appurtenances. Given a family of a certain size and this is enough, given a larger family and it may be too little; but of the many writers on the subject few have considered the cost. At pre-war prices such a dwelling would have cost not less than £275 to build. At present prices it is very doubtful if £450 would provide it. If that sum sufficed the rent would have to be £36 per annum, or, say, 14s. per week; that is the nearest round sum. This is 8 per cent. gross, not too large a sum for the man who by his self-denial and saving finds the money necessary to build the house, for out of it he has to provide reasonable interest, repairs, insurance, depreciation, and stand the risk of times of emptiness.

### Co-operation to the Rescue.

If the calculation be right that a man should pay one-eighth of his income for rent, the tenant should have a certain income of £5 12s. a week. The wealth of Great Britain is enormous, but it is doubtful if it is possible to pay to every wage-earner such a minimum wage as this without bankruptcy ensuing. It is certain private persons will not build houses and let them at a rate that represents a loss. It is not advisable for the State to do so. The difficulty comes because the standard of housing has gone high. The one-roomed daub and wattle was succeeded by the two-roomed thatched cottage, and that by a better, till tidy little houses have been evolved as the standard of to-day. And the State is insisting on the standard. No man is allowed to live in a log cabin or a frame house such as he who breaks the prairie in Canada, or goes lumbering in British Columbia, or takes work on a station in Australia would inhabit, till by his work and self-denial he had accumulated the capital to build better. If it be not possible for every man to have all the accommodation and convenience of a really good-class house to his own private use, it is worth considering if he could not have them by co-operation. In a great city every man cannot have a charming garden, but collectively all men have, and enjoy, gardens and parks that the richest may envy—Kensington Gardens, the beautiful parks of Sheffield, miles of them, and of many other great cities.

### The Poor Man's Mansion.

By a system of collective housing might it not be possible to provide spacious and noble accommodation for all men earning a wage at a rent within the means? Instead of many small houses in undistinguished streets, imagine great mansions of tasteful design, the main entrances opening into spacious halls in which a hundred persons can sit comfortably. Beyond the hall a great corridor, and off it the women's rooms, the men's rooms, the writing-rooms, the restaurant, and its great kitchens and sculleries attached, the silent rooms, the reading-rooms, a card-room, a games-room, and great nurseries for the children. Lifts and great stairways take the inhabitants to upper floors on which are many rooms of various sizes, mostly small, some single, some in pairs, some in sets of three or four opening one into another as well as into the common corridors. Central heating, electric light, bathrooms.

### Cost and Working.

The scale calculated for 1,000 persons, each renting so many rooms as each could afford, and having the use of the common rooms, the common heating, the common cooking, the common water supply. What would such great co-operative homes cost to build? Certainly it should be far less per head for equally good accommodation than the cost of six- and seven-roomed houses for families individually. Probably the rent paid for as many bedrooms as would be obtained for the same sum at the economic rent of a small house would include, besides the use of the common rooms, the expenses of heating, lighting, water supply, and cooking. Meals provided on a restaurant system in the common dining-halls, and a small sum per child to nurses in the common nurseries, would free the mothers from the main household cares and enable such as wished to be wage-earners. In the building itself there would be work for many of the wives living in it. Cooks and scullery-maids and nurses, and women to keep clean and tidy the common rooms are obvious requirements. Such would be paid a wage, and would pay for such private accommodation as each required.

### The Hotel Habit.

There is no need that the building should be a profit-making concern beyond a moderate interest on capital. All profits beyond, say, 6 per cent., after provision for depreciation, might be divided in proportion to their weekly payments amongst the inhabitants of the co-operative home. Such an idea as this is not in accordance with English habits, but equally a few dozen decades ago it might have been said that living in great cities was foreign to English habits. But to be urban has become the English habit, and if urban a population cannot live as if in primitive and rural surroundings. Habits must alter to suit environment. If the choice be between stuffy little expensive houses in mean streets, and great common homes on the principle of great hotels such as are even now the homes of great numbers of well-to-do persons of the middle classes, the latter may be the better to choose.

